# Development and evaluation of virtual simulation games to increase the confidence and self-efficacy of healthcare learners in vaccine communication, advocacy, and promotion

**DOI:** 10.1186/s12909-024-05169-9

**Published:** 2024-02-25

**Authors:** Emily J. Doucette, Madison M. Fullerton, Margaret Pateman, Alyssa Lip, Sherilyn K. D. Houle, James D. Kellner, Jenine Leal, Shannon E. MacDonald, Deborah McNeil, Jane Tyerman, Marian Luctkar-Flude, Sandra Davidson, Cora Constantinescu

**Affiliations:** 1 Department of Pediatrics, Cumming School of Medicine, University of Calgary, Calgary, AB Canada; 2 Faculty of Nursing, University of Calgary, Calgary, AB Canada; 3 19 to Zero Inc, Rocky Mountain House, AB, Canada; 4 Division of Respirology, Department of Medicine, University of Calgary, Calgary, AB Canada; 5 School of Pharmacy, University of Waterloo, Waterloo, ON Canada; 6 Alberta Children’s Hospital Research Institute, University of Calgary, Calgary, AB Canada; 7 Department of Community Health Sciences, Cumming School of Medicine, University of Calgary, Calgary, AB Canada; 8 Department of Microbiology, Immunology and Infectious Diseases, University of Calgary, Calgary, AB Canada; 9 O’Brien Institute for Public Health, University of Calgary, Calgary, AB Canada; 10 Infection Prevention and Control, Alberta Health Services, Calgary, AB Canada; 11 Antimicrobial Resistance - One Health Consortium, University of Calgary, Calgary, AB Canada; 12 Real World Evidence Consortium, University of Calgary, Calgary, AB Canada; 13 Faculty of Nursing, University of Alberta, Edmonton, AB Canada; 14 Alberta Health Services, Maternal Newborn Child and Youth Strategic Clinical Network, Calgary, AB Canada; 15 School of Nursing, University of Ottawa, Ottawa, ON Canada; 16 Canadian Alliance of Nurse Educators using Simulation (CAN-Sim), Kingston, ON Canada; 17 School of Nursing, Queen’s University, Kingston, ON Canada; 18 Pediatric Infectious Diseases, Department of Pediatrics, Cumming School of Medicine, University of Calgary, Calgary, AB Canada

**Keywords:** Virtual simulation, Simulation games, Healthcare learner, Vaccine hesitancy, Communication, Evaluation

## Abstract

**Background:**

Although healthcare providers (HCPs) are the most trusted source of vaccine information, there is a paucity of easily accessible, multidisciplinary educational tools on vaccine communication for them. Virtual simulation games (VSGs) are innovative yet accessible and effective tools in healthcare education. The objectives of our study were to develop VSGs to increase HCP confidence and self-efficacy in vaccine communication, advocacy, and promotion, and evaluate the VSGs’ effectiveness using a pre-post self-assessment pilot study.

**Methods:**

A multidisciplinary team of experts in medicine, nursing, pharmacy, and simulation development created three VSGs for HCP learners focused on addressing conversations with vaccine hesitant individuals. We evaluated the VSGs with 24 nursing students, 30 pharmacy students, and 18 medical residents who completed surveys and 6-point Likert scale pre-post self-assessments to measure changes in their confidence and self-efficacy.

**Results:**

There were no significant differences in baseline confidence and self-efficacy across the three HCP disciplines, despite varied levels of education. Post-VSG confidence and self-efficacy (median: 5) were significantly higher than pre-VSG (median: 4–5) for all three HCP disciplines (*P* ≤ 0.0005), highlighting the effectiveness of the VSGs. Medical residents reported significantly lower post-VSG confidence and self-efficacy than nursing and pharmacy learners despite completing the most significant amount of education.

**Conclusions:**

Following the completion of the VSGs, learners in medicine, nursing, and pharmacy showed significant improvement in their self-assessed confidence and self-efficacy in holding vaccine conversations. The VSGs as an educational tool, in combination with existing clinical immunization training, can be used to increase HCP confidence and engagement in vaccine discussions with patients, which may ultimately lead to increased vaccine confidence among patients.

**Supplementary Information:**

The online version contains supplementary material available at 10.1186/s12909-024-05169-9.

## Background

Prior to the COVID-19 pandemic, vaccine hesitancy (the delay in acceptance or refusal of vaccination despite vaccine availability) [1] was named one of the top ten global health concerns by the World Health Organization [2]. Following the pandemic, global vaccine hesitancy continues to be a significant factor preventing the public from vaccinating against infectious diseases such as COVID-19 [3, 4]. As of December 2023, only 15.0% of Canadians are up to date with the recommended COVID-19 immunizations [5]. Further, childhood routine immunization rates have also decreased both in Alberta, Canada [6] and globally [4, 7] since the pandemic began. It is well-cited that healthcare providers (HCPs) are patients’ most trusted source of health and immunization information, suggesting that conversations with HCPs about vaccines may be an effective way to address vaccine hesitancy [8–10].

Although HCPs can address vaccine hesitancy, their willingness to recommend immunization for their patients is dependent on their knowledge of vaccine effectiveness and safety in addition to their personal feelings towards vaccination [8]. It is essential that all HCPs feel confident in themselves and maintain a professional sense of self-efficacy when engaging in vaccine discussions. While confidence generally describes “a strong self-perceived belief” and can be both positive or negative [11], self-efficacy describes “an individual’s belief in their capacity to execute behaviours necessary to produce specific outcomes” [12, 13]. A recent scoping review by our team examined whether educational interventions existed for HCPs on how to effectively engage in vaccine conversations with patients [14]. The review identified that current interventions are not easily accessible, are targeted towards medical learners (e.g., medical students, residents, physicians, etc.) and are not inclusive of other professionals that also play a key role in immunization distribution and uptake, such as nurses and pharmacists.

Virtual simulation games (VSGs) are a form of digital learning and have been used in clinical nursing education for many years [15], but their development and use have become increasingly common since the COVID-19 pandemic restricted in-person learning [16]. The Canadian Alliance of Nurse Educators Using Simulation (CAN-Sim) is a leading non-profit organization in simulation education and research with extensive experience developing VSGs for HCPs on various health topics (18). VSGs can provide additional decision-making opportunities for participants to complement in-person simulation that often occurs in large group settings, as well as increased accessibility by learners worldwide [16]. VSGs can also provide psychological safety and lessen fear, anxiety, and embarrassment in new learners who may worry about making mistakes in front of fellow students and instructors [16–18].

When considering interventions to address vaccine hesitancy, it is important to consider the framing of the conversation and the information being presented to a patient. Patients were more likely to accept vaccine recommendations from a HCP if they used presumptive statements (e.g., “Today you will receive two vaccinations, correct?”) rather than participatory (e.g., “Are we going to vaccinate today?”) (21). The PrOTCT Framework provides HCPs with a structure to discuss vaccinations with patients and build trust by **P**resuming the patient will vaccinate, **O**ffering to share knowledge and personal experiences with vaccines, **T**ailoring recommendations to address patients’ specific health **C**oncerns, and **T**alking through a specific plan for when and where to get vaccinated [19]. Taken together, presumptive statements and the evidence-based PrOTCT Framework could serve as the basis for creating evidence-informed educational materials to better support HCPs in discussing vaccines with their patients.

Given the paucity of accessible and multidisciplinary educational tools alongside the benefits of virtual simulation, the objectives of this study were: to (1) develop three VSGs to increase the confidence and self-efficacy of HCP learners in vaccine communication, advocacy, and promotion through the use of presumptive statements and the PrOTCT Framework, and (2) evaluate the VSGs using a pre- and post-intervention self-assessment to measure their perceived effectiveness in increasing HCP vaccine communication confidence and self-efficacy.

## Methods

A diverse team of subject matter experts in the areas of medicine, nursing, and pharmacy came together to support and guide the development of three VSGs based on common evidence-based vaccine hesitancy topics encountered in clinical practice. Each VSG was designed to take approximately 20 minutes to complete. The team met virtually in February and March 2022 via the Zoom platform (Zoom Video Communication, San Jose, California, USA) to design the VSGs with leadership from and templates provided by CAN-Sim.

### Virtual simulation game development

The VSGs were developed through a series of virtual workshops led by CAN-Sim experts using the CAN-Sim VSG design framework (18), with a focus on incorporating presumptive statements (21) and the PrOTCT model [19] as the communication framework for the games. The VSGs were designed to be discipline and knowledge agnostic to ensure that any of the three healthcare disciplines could complete the intervention regardless of their practice setting or specific knowledge about vaccine administration, side effects, and ingredients. Based on the scoping review conducted by our team [14] as well as input from subject matter experts in the areas of medicine, nursing, and pharmacy, we chose the following topics related to vaccine hesitancy to be covered in each game: how to have a conversation with a patient expressing hesitancy around receiving a booster/completing a vaccine series (VSG1); how to support a patient who minimizes the risk of disease while maximizing the risk of the vaccine (VSG2); and how to foster personal resilience, maintain a professional sense of self-efficacy, and prevent burnout and moral distress during challenging patient interactions (VSG3).

Once a case summary was drafted for each scenario, the first step of the VSG development process was the creation of learning outcomes and indicators (Additional file 1), followed by the creation of self-assessment rubrics using Likert scales (Additional file 2). Next, using the CAN-Sim Decision Point Map template (Additional file 3), decision points were created based on the established learning outcomes to outline the flow of the games. Each decision point consists of a critical thinking question with one correct response and two responses that were not correct or not the best answer. The evidence-based rationale for each decision point was determined by the content experts from each discipline and was kept consistent through each VSG. The filming scripts were then written in teams consisting of nurses, pharmacists, and physicians, as well as learners from these disciplines to determine the dialogue between characters based on the learning outcomes and decision points. The scripts were reviewed by key stakeholders in the community, including practicing clinicians and learners in the three disciplines, and two individuals (one who identified as male aged 65–75 and one as female aged 55–65 who had a dependent son with medical complexity) from the public who identified as vaccine hesitant. After completion of the peer review and incorporation of feedback, the VSGs were filmed in person with actors. The games were assembled by the CAN-Sim team using Articulate Storyline 3 and Rise software and made available through an online open-access website [20].

### Target audience and pilot participant recruitment

The target audience for the VSGs were HCP learners in medicine, nursing, and pharmacy. However, in efforts to pilot the games before broader dissemination, we recruited medical residents from the specialties of Internal Medicine (IM), Family Medicine (FM), Obstetrics and Gynecology (OBGYN), Pediatrics (Peds), Emergency Medicine (EM), and undergraduate nursing students in their third and fourth year from the University of Calgary, while undergraduate pharmacy students in their second, third, and fourth year were recruited from the University of Waterloo. These demographics were identified as the most likely to have had prior clinical experience with vaccine conversations as opposed to medical students and early nursing and pharmacy learners. Further, the scoping review [14] identified many existing educational interventions targeted towards medical students, so residents were chosen as the focus of this project.

Informed consent was obtained prior to study participation. Participants were offered an electronic gift card for their time spent completing the VSGs in the amount of CAD $25 per VSG completed up to CAD $75 total. The target sample size was calculated to be 30 participants from each discipline (medicine, nursing, and pharmacy) to participate in the pilot evaluation of the VSGs. Sample size targets were based on statistical analysis using G*Power [21]. Assuming the intervention would increase the perceived confidence in discussing vaccine hesitancy with a one-sided t-test, a medium effect size of 0.5, power of 0.8, and type I error probability of 0.05, the calculated minimum sample size per group was 28.

### Virtual simulation game evaluation using pre- and post-intervention self-assessments

Prior to and after completing the three VSGs, participants were asked to self-assess their confidence and self-efficacy using 6-point self-assessment Likert-based scales for each VSG ranging from 1 (least amount of confidence/self-efficacy) to 6 (most amount of confidence/self-efficacy) (Additional file 2). The three self-assessment scales were designed using CAN-Sim templates and were reviewed by a team of experts to ensure content was appropriate.

### Data analysis

Demographic factors, including participants’ age, gender, year of program, medical specialty (residents only), and questions about whether participants had experience learning about vaccine conversations, were collected in the pre-intervention survey (Additional file 4). Differences in demographic factors between HCP disciplines were assessed using Fisher’s exact test and reported significant if *P* < 0.05.

Statistical analysis of pre-post self-assessment responses was conducted on each of the VSGs independently. Individual Likert item scores for questions were stratified into two evaluation categories, confidence and self-efficacy, for each VSG (VSG 1 had three confidence questions and two self-efficacy questions, VSG 2 had three confidence questions and three self-efficacy questions, and VSG 3 had two confidence questions and two self-efficacy questions) (Additional File 2). Non-parametric testing methods were used as normality assumptions and target sample sizes were not met, and significant skew was identified visually and confirmed statistically using the Shapiro-Wilk test. A one-sided Wilcoxon matched-pairs signed rank test using Pratt’s method was used to compare the paired difference between median scores in pre- and post-assessments, and a Kruskal-Wallis test with Dunn’s multiple comparison test was used to compare self-assessment scores across HCP disciplines (significance reported based on an alpha value of 0.05). Unpaired responses (missing a pre- or post-survey response) were included for descriptive statistics and excluded during paired analyses. Data was analyzed using R 1.1.463, and figures were created using GraphPad Prism 9.2.0.

## Results

A total of 81 participants provided consent to participate in the study; however, nine were excluded from analyses due to more than one missing or incomplete self-assessment (four pharmacy and four medical learners) or being unable to confirm enrollment in a HCP training program (one nursing learner). Data from 72 participants, including 24 nursing, 30 pharmacy, and 18 medicine learners, were included in the final analysis.

There were no significant differences in participants’ gender across the three disciplines, however, medical learners were significantly older than nursing and pharmacy learners (Table 1). All pharmacy and medical learners reported having a previous vaccine conversation with patients, compared with only 70.8% of nursing learners (Table 1). Whether or not participants learned about how to have vaccine conversations with patients in their program also differed significantly by discipline (*P* < 0.001) (Table 1), and the most common setting participants learned to have conversations was in theory and/or coursework.

**Table 1:** Participant demographics from pre-intervention survey (P-values calculated with Fisher’s exact test)

	Nursing Students (*N* = 24)	Pharmacy Students (*N* = 30)	Medical Residents (*N* = 18)	Total (*N* = 72)	P-value
**Age (n, %)**					< 0.0001
18–25	15 (62.5)	22 (73.3)	2 (11.1)	39 (54.2)	
26+	7 (29.2)	7 (23.3)	15 (83.3)	29 (40.3)	
Unknown	2 (8.3)	1 (3.3)	1 (5.6)	4 (5.6)	
**Gender (n, %)**					0.3884
Female	19 (79.2)	23 (76.7)	13 (72.2)	55 (76.4)	
Male	4 (16.7)	7 (23.3)	5 (27.8)	16 (22.2)	
Non-binary	1 (4.2)	0 (0.0)	0 (0.0)	1 (1.4)	
**Year of HCP training program (n, %)**					< 0.0001
1st	1 (4.2)	0 (0.0)	6 (33.3)	7 (9.7)	
2nd	0 (0.0)	3 (10.0)	8 (44.4)	11 (15.3)	
3rd	9 (37.5)	21 (70.0)	1 (5.6)	31 (43.1)	
4th	14 (58.3)	5 (16.7)	3 (16.7)	22 (30.6)	
Other (Graduated)	0	1 (3.3)	0 (0.0)	1 (1.4)	
**Medical Specialty (n, %)** ^**1**^					N/A
FM			8 (44.4)	8 (44.4)	
OBGYN			1 (5.6)	1 (5.6)	
Peds			7 (38.9)	7 (38.9)	
PHPM			2 (11.1)	2 (11.1)	
**Have you ever had a vaccine conversation with a patient? (Not necessarily about VH) (n, %)**					0.0003
Yes	17 (70.8)	30 (100.0)	18 (100.0)	65 (90.3)	
No	7 (29.2)	0 (0.0)	0 (0.0)	7 (9.7)	
**Did you learn about how to have vaccine conversations with patients in your program? (n, %)**					< 0.0001
Yes	4 (16.7)	26 (86.7)	9 (50.0)	39 (54.2)	
No	13 (54.2)	2 (6.7)	7 (38.9)	22 (30.6)	
Unsure	7 (29.2)	2 (6.7)	2 (11.1)	11 (15.3)	
**If yes, where you learned about vaccine conversations (select all that apply) (n)**					N/A
Theory/coursework	1	15	4	20	
Lab/simulation setting	1	10	0	11	
Clinical practice	1	4	4	9	
Workshop	0	2	1	3	

^1^FM=family medicine, OBGYN = obstetrics and gynecology, Peds = pediatrics, PHPM = public health and preventative medicine

**Fig. 1:**
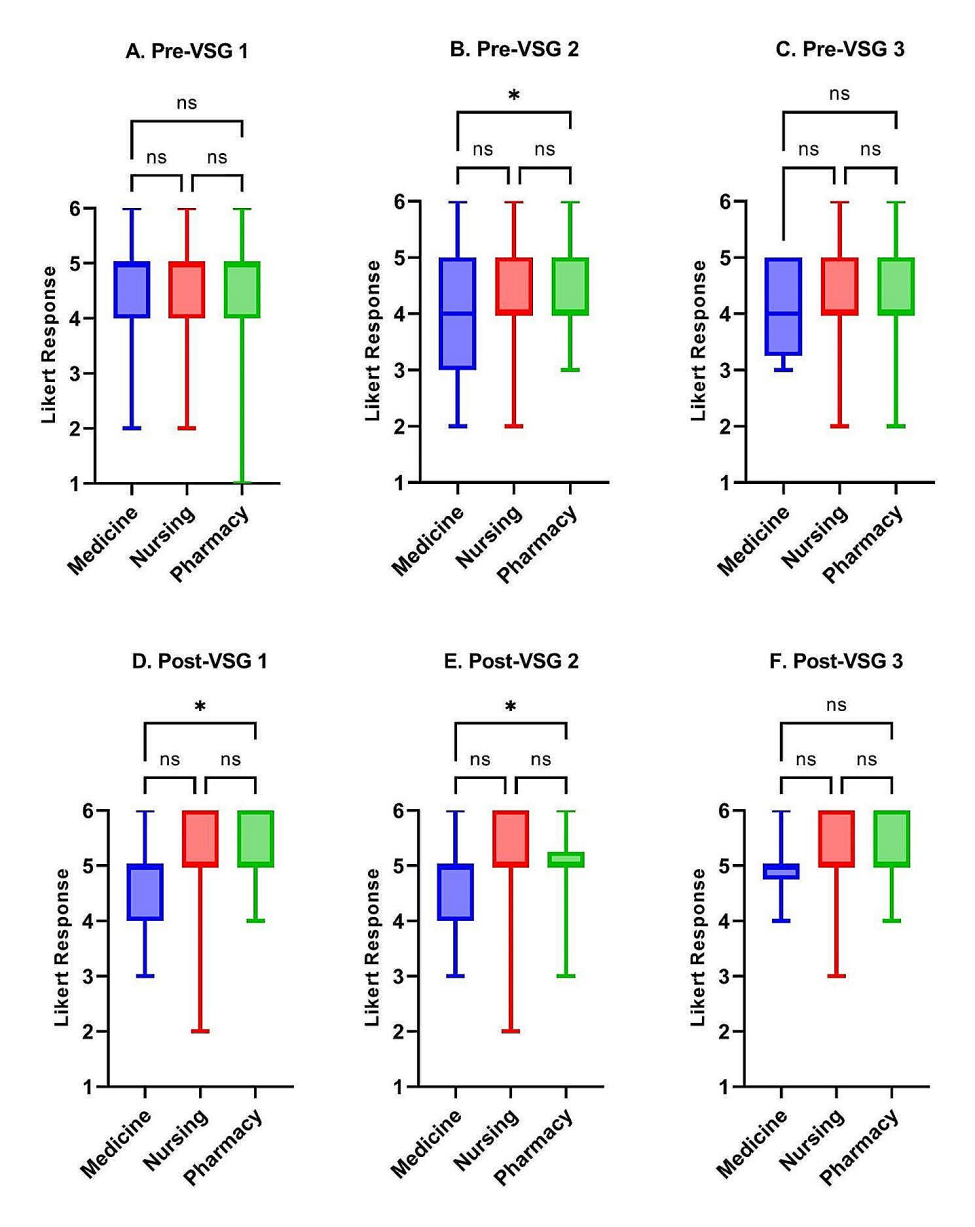
Confidence score comparisons between HCP learner disciplines (medicine residents, nursing students, and pharmacy students) for each pre- and post-VSG self-assessment. (ns = *P* > 0.05, * = *P* ≤ 0.05, ** = *P* ≤ 0.01, *** = *P* ≤ 0.001)

**Fig. 2:**
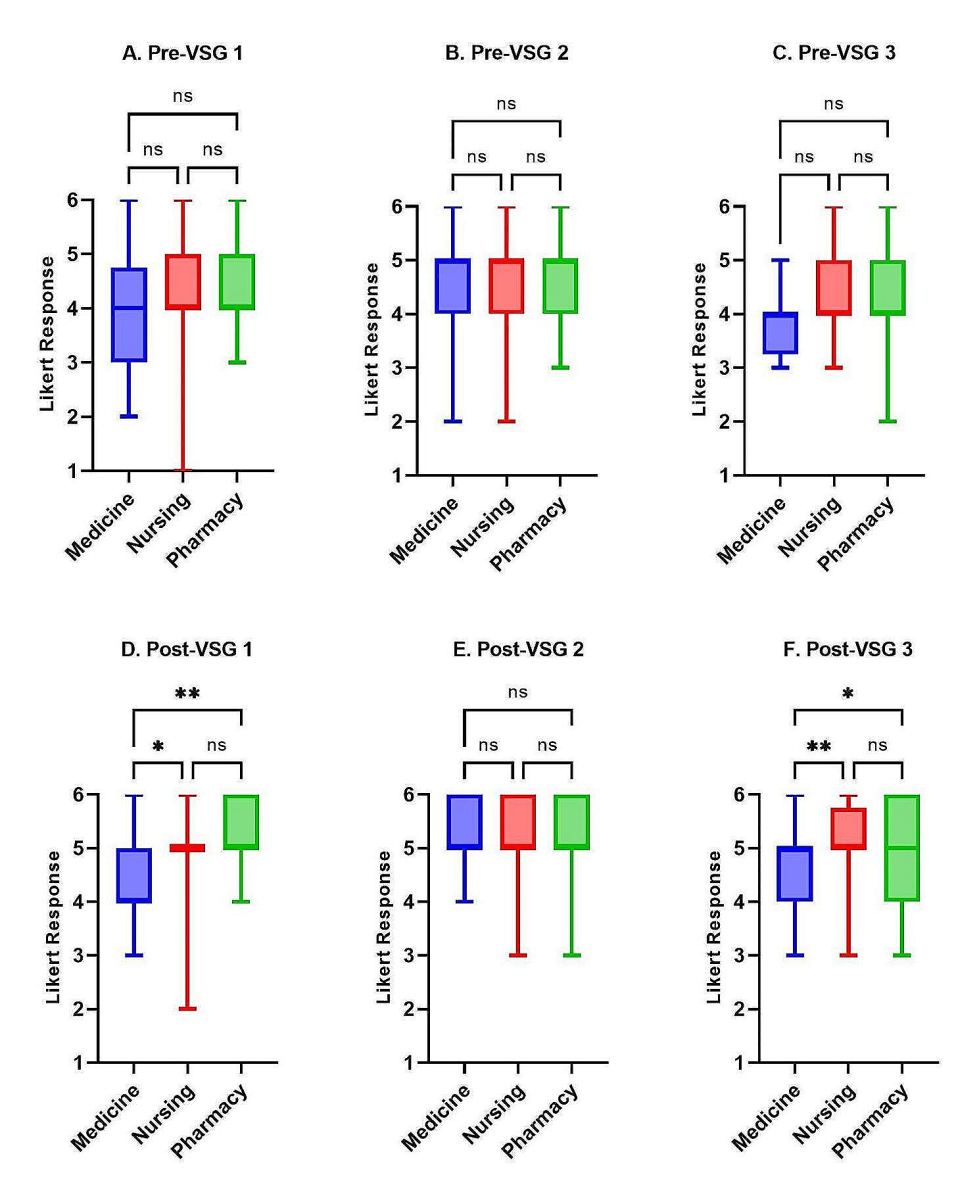
Self-efficacy score comparisons between HCP learner disciplines (medicine residents, nursing students, and pharmacy students) for each pre- and post-VSG self-assessment. (ns = *P* > 0.05, * = *P* ≤ 0.05, ** = *P* ≤ 0.01, *** = *P* ≤ 0.001)

No significant differences in baseline confidence or self-efficacy were identified across the three disciplines on VSG1 and VSG3 (median score = 4); however, medical and pharmacy learner responses differed significantly in self-confidence before VSG2 (*P* < 0.05) (Figs. 1A-C and 2A-C). Medical learners reported significantly lower confidence and self-efficacy than nursing and pharmacy learners on all three post-intervention self-assessments, with the exception of post-VSG2 self-efficacy which had no differences between disciplines (Figs. 1D-F and 2D-F).

**Fig. 3:**
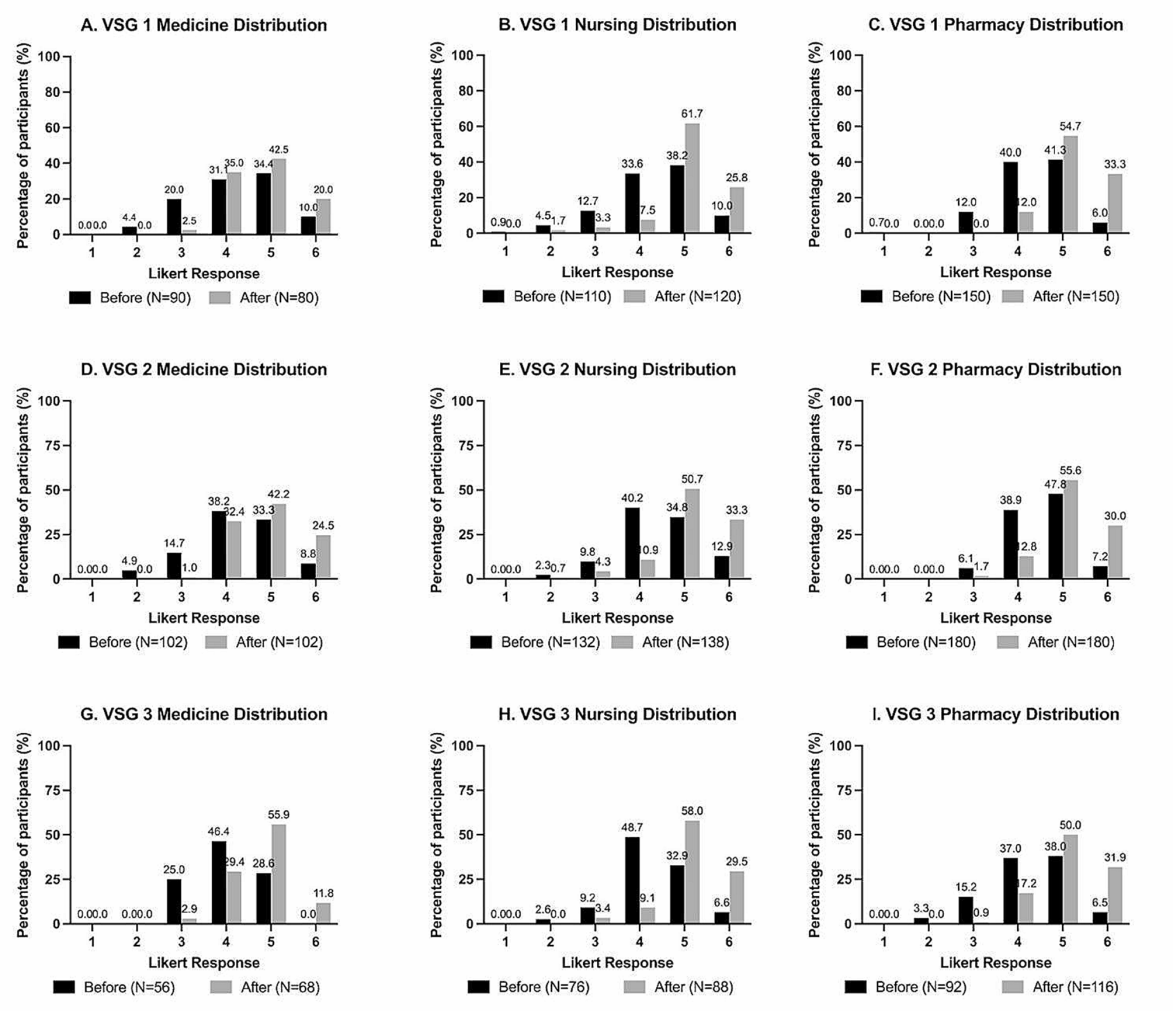
Pre- and post-intervention self-assessment response distributions. Likert scale responses ranged from 1 (least amount of confidence/self-efficacy) to 6 (most amount of confidence/self-efficacy)

All pre- and post-intervention self-assessment scores were non-normally distributed and skewed to the upper end of the 6-point Likert scales (Fig. 3, Additional file 2). The median response for confidence and self-efficacy pre-self-assessments ranged from 4 to 5, while the median response for post-self-assessments was 5 (with the exception of the VSG1 self-efficacy score by medical learners) (Table 2). The overall post-intervention scores for both confidence and self-efficacy questions were significantly higher than the pre-intervention scores across all three HCP disciplines and for all three VSGs (Table 2).

**Table 2:** Self-assessment response overview per VSG by HCP discipline. VSG1: 3 confidence questions and 2 self-efficacy questions, VSG2: 3 confidence questions and 3 self-efficacy questions, and VSG3: 2 confidence questions and 2 self-efficacy questions

VSG 1					
Discipline	Attribute Assessed	Self-Assessment (pre/post)	Participant responses (n)	Median Response (range)	P value
Medicine	Confidence	Pre	48	5 (2–6)	0.0005
		Post	48	5 (3–6)	
	Self-efficacy	Pre	32	4 (2–6)	< 0.0001
		Post	32	4 (3–6)	
Nursing	Confidence	Pre	66	5 (2–6)	< 0.0001
		Post	66	5 (2–6)	
	Self-efficacy	Pre	44	4 (1–6)	< 0.0001
		Post	44	5 (2–6)	
Pharmacy	Confidence	Pre	90	5 (1–6)	< 0.0001
		Post	90	5 (4–6)	
	Self-efficacy	Pre	60	4 (3–6)	< 0.0001
		Post	60	5 (4–6)	
**VSG 2**					
Discipline	Attribute Assessed	Self-Assessment (pre/post)	Participant responses (n)	Median Response (range)	P value
Medicine	Confidence	Pre	51	4 (2–6)	< 0.0001
		Post	51	5 (3–6)	
	Self-efficacy	Pre	51	5 (2–6)	< 0.0001
		Post	51	5 (4–6)	
Nursing	Confidence	Pre	66	4 (2–6)	< 0.0001
		Post	66	5 (2–6)	
	Self-efficacy	Pre	66	5 (2–6)	< 0.0001
		Post	66	5 (3–6)	
Pharmacy	Confidence	Pre	90	4 (3–6)	< 0.0001
		Post	90	5 (3–6)	
	Self-efficacy	Pre	90	5 (3–6)	< 0.0001
		Post	90	5 (3–6)	
**VSG 3**					
Discipline	Attribute Assessed	Self-Assessment (pre/post)	Participant responses (n)	Median Response (range)	P value
Medicine	Confidence	Pre	28	4 (3–5)	0.0002
		Post	28	5 (4–6)	
	Self-efficacy	Pre	28	4 (3–6)	0.0001
		Post	28	5 (3–6)	
Nursing	Confidence	Pre	38	4 (2–6)	< 0.0001
		Post	38	5 (3–6)	
	Self-efficacy	Pre	38	4 (3–6)	< 0.0001
		Post	38	5 (3–6)	
Pharmacy	Confidence	Pre	46	4 (2–6)	< 0.0001
		Post	46	5 (4–6)	
	Self-efficacy	Pre	46	4 (2–6)	< 0.0001
		Post	46	5 (4–6)	

Although most paired participant scores improved after each game (57.5% of all responses for VSG1, 55.2% for VSG2, 53.3% for VSG3), some scores did not change (33.6% of all responses for VSG1, 39.5% for VSG2, 24.6% for VSG3), while a small amount decreased (3.3% of all responses for VSG 1, 3.8% for VSG2, 4.4% for VSG3). 5.6%, 1.4%, and 17.6% of responses for VSG1, VSG2, and VSG3, respectively, were unpaired due to missing or incomplete self-assessments.

## Discussion

In this study, we designed and developed three VSGs to increase the confidence and self-efficacy of HCP learners in vaccine communication, advocacy, and promotion and evaluated their efficacy with participants in medicine, nursing, and pharmacy. Learners in all three disciplines reported significantly improved self-confidence and self-efficacy scores in the post-VSG self-assessments for all three VSGs. These findings support the introduction of VSGs into the clinical training of all levels of HCPs from undergraduate to post-graduate who may discuss vaccines with patients. Several unique strengths of the VSGs include their multidisciplinary development process, which utilized the engagement of multiple stakeholders, collaboration with CAN-Sim, and the discipline and knowledge-agnostic content (in comparison to most other online learning modules that often target a specific vaccine or a single discipline).

Despite varied levels of education across the three HCP learner disciplines, participants reported similar baseline scores on all three pre-VSG self-assessments, suggesting that the variable amounts of immunization-related training and clinical experiences in different HCP programs previously identified [22] does not result in any differences in HCP learner confidence or self-efficacy. Ultimately, HCPs are most likely to improve their confidence in communication skills through repeated practice with patients, and simulations are an effective way to emulate real patient scenarios. They offer a low-stakes environment where learners can try new skills, receive immediate feedback, and learn from mistakes [16–18].

The use of pre-post measurements with Likert scale assessments introduces a potential for initial overconfidence bias and post-test overcorrection due to the Dunning-Kruger effect [23, 24], where a lack of knowledge can cause someone to overestimate their skills. This was likely observed in the small proportions of participants whose scores did not change or decreased from pre- to post-test. Interestingly, medical learners consistently reported significantly lower levels of self-confidence in the post-VSG self-assessments compared to participants in nursing and pharmacy despite completing the most years of medical education. This may be a result of the Dunning-Kruger effect, or it may be possible that with greater amounts of training and responsibility, medical learners experience increased feelings of patient care ownership (PCO). PCO, or feelings of accountability for patients when in charge of clinical decision-making [25, 26], has been shown to increase as residents’ seniority increases [25, 27]. Therefore, residents who feel greater levels of PCO and who fear hindering the therapeutic relationship with long term patients could result in overall lower perceived self-confidence.

## Limitations

This study has several limitations. Each survey had some unpaired pre- or post-assessment responses. VSG 3 in particular had the largest percentage of missing pre-VSG responses, suggesting problems with the Qualtrics survey website may have prevented some responses from being saved. Due to the challenge of recruiting participants from busy HCP training programs, we relied on convenience sampling and recruited a smaller number of participants than initially anticipated. This reduces our statistical power, increases the likelihood that learners with lower initial levels of self-confidence were excluded, and decreases our ability to generalize our findings to the broader population. To lessen the impact of scheduling conflicts resulting in participant dropouts, we offered flexible options to those participating to maximize completion rates. Additionally, the use of ordinal Likert scales for self-assessments and a pre-post-test study design limits statistical analysis and introduces potential social desirability bias [28]. We attempted to mitigate this by emphasizing the anonymity of the assessments to encourage honest self-reflection. Future studies may benefit from using a continuous scale assessment and/or a randomized control trial design to increase generalizability. Face validity of the outcome measure of confidence and self-efficacy was established however further construct validity and reliability testing would be strengthen its use. Lastly, we recruited participants from only one institution per profession, although baseline scores were similar across all professions which decreases the likelihood that inter-institutional differences in how vaccine-related content is taught impacted our results.

## Conclusions

Our virtual simulation modules significantly improved HCP learners’ confidence in holding challenging vaccine conversations with patients. Based on our findings, we recommend the development and use of VSGs for both HCP education programs and accredited continuing education programs as they are easily accessible and can be used by any HCP learner or practitioner. Further, these learning modules can easily be expanded to add additional content, further assessments, and practice material on many different vaccine hesitancy situations. In combination with existing didactic immunization training, these virtual simulation modules will complement HCPs existing knowledge and provide useful tools and skills to increase the likelihood they engage in conversations with those who are vaccine hesitant. Such interventions will continue to strengthen the patient-provider relationship, build trust, and provide support to both HCPs and the public in vaccination decisions.

### Electronic supplementary material

Below is the link to the electronic supplementary material.

Supplementary Material 1: Additional file 1. Learning outcomes and indicators example (VSG 1).

Supplementary Material 2: Additional file 2. VSG self-assessment rubrics (VSG 1, VSG 2, VSG 3).

Supplementary Material 3: Additional file 3. Decision point map and rationale example (VSG 1).

Supplementary Material 4: Additional file 4. Pre- and post-intervention surveys.

## Data Availability

The datasets used and/or analyzed during the current study are available from the corresponding author on reasonable request.
