# Supplementary material for: Development and evaluation of virtual simulation games to increase the confidence and self-efficacy of healthcare learners in vaccine communication, advocacy, and promotion
Source: BMC Med Educ. 2024 Feb 25;24:190. doi: 10.1186/s12909-024-05169-9 (PMC10895736; doi:10.1186/s12909-024-05169-9)
Supplement: Supplementary file 1 — Supplementary Material 1: Additional file 1. Learning outcomes and indicators example (VSG 1). [file 12909_2024_5169_MOESM1_ESM.docx]

Additional file 2. VSG Self-Assessment Rubrics

1. VSG 1 - Vaccine Hesitancy - Booster Assessment Rubric

| **Learning Outcome** | **Confidence/Self-efficacy Indicators** |  |  |  |
| --- | --- | --- | --- | --- |
| **Which statement best describes how confident you are regulating your emotions before, during and after a clinical encounter to promote a therapeutic relationship with patients?** | - I focus on problem-solving - I can approach situations with an open mindset - I can identify and recognize specific triggers - I am aware of what I am feeling - I can identify the causes of my emotions - I can identify a ‘safe space’ when emotions are heightened | **I am fully confident that I can** prioritize personal emotional regulation before, during and after a clinical encounter. | **I am somewhat confident that I can** prioritize personal emotional regulation before, during and after a clinical encounter. | **I am not confident that I can** prioritize personal emotional regulation before, during and after a clinical encounter. |
| Comments: | Likert Scale 6 5 4 3 2 1 | | | |
| **Which statement best describes how confident you are in developing rapport in a healthcare setting to build an ongoing relationship with a patient?** | - I can create a safe space using verbal and non-verbal communication skills - I can engage in an honest conversation with patients - I know how to use active listening skills - I can establish a common ground with patients | **I am fully confident** in my ability and skills to develop a rapport with a patient to establish/build upon a therapeutic relationship. | **I am somewhat confident** in my ability and skills **to** develop a rapport with a patient to establish/build upon a therapeutic relationship. | **I am not confident** in my ability and skills to develop a rapport with a patient to establish/build upon a therapeutic relationship. |
| Comments: | Likert Scale 6 5 4 3 2 1 | | | |
| **Which statement best describes how confident you are making presumptive statements around vaccination during a clinical encounter with a patient who has not completed a vaccine series to reinforce positive behaviours?** | - I assume a person wants to optimize their personal health outcomes - I assume a person will choose to get immunized - I can give strong recommendations - I can make the presumptive statement in a relevant and appropriate manner - I am respectful, calm, and non-judgmental | **I am fully confident** using these techniques when making presumptive statements. | **I am somewhat confident** using these techniques when making presumptive statements. | I **am not confident** using these techniques when making presumptive statements. |
| Comments: | Likert Scale 6 5 4 3 2 1 | | | |
| **Which statement best describes your ability to recognize vaccine hesitancy profiles**  **during a routine clinical encounter to determine approaches to understanding reasons for not continuing with vaccine series?** | - I am able to review client vaccine needs - I am able to explore patients’ reasons for vaccine hesitancy - I am able to identify specific patient concerns | **I am consistently** able to recognize vaccine hesitancy profiles to identify a patient’s priority concerns. | I **am sometimes** able to recognize vaccine hesitancy profiles to identify a patient’s priority concerns. | **I am rarely able to** recognize a vaccine hesitancy profile to identify the patient’s priority concerns. |
| Comments: | Likert Scale 6 5 4 3 2 1 | | | |
| **Which statement best describes your ability to support decision-making for patients who have not completed a vaccine series to develop a personalized and achievable plan for vaccination?** | - I support patients to recognize and acknowledge risks and benefits of each option - I am able to support clients’ values and preferences - I am able to address patient needs - I am able to provide additional resources if requested | **I am consistently able to** support decision-making regarding vaccines with a patient who has not completed a vaccine series. | **I am sometimes able to** support decision-making regarding vaccines with a patient who has not completed a vaccine series. | **I am rarely able to** support decision-making regarding vaccines with a patient who has not completed a vaccine series. |
| Comments | Likert Scale 6 5 4 3 2 1 | | | |

1. VSG 2 - Responding to Vaccine Hesitancy Assessment Rubric

| **Learning Outcome** | **Confidence/Self-efficacy Indicators** |  |  |  |
| --- | --- | --- | --- | --- |
| **Which statement best describes your ability to recognize your own emotions before, during and after the clinical encounter to provide a therapeutic relationship with the patient?** | - I focus on problem-solving - I am able to approach situations with an open mindset - I am able to identify and recognize triggers - I am aware of what you I am feeling - I am able to identify the causes of my emotions - I am able to identify a ‘safe space’ when emotions are heightened | **I am consistently able to** recognize my own emotions before, during and after a clinical encounter | **I am sometimes able to** recognize my own emotions before, during and after a clinical encounter | **I am rarely able to** recognize my own emotions before, during and after a clinical encounter |
| Comments | Likert Scale 6 5 4 3 2 1 | | | |
| **Which statement best describes your ability to develop rapport in a healthcare setting to build an ongoing relationship with the patient?** | - I am able to create a safe space using verbal and non-verbal communication skills - I am able to engage in an honest conversation - I use active listening skills - I am able to establish a common ground | **I am consistently able to** develop a rapport in a healthcare setting | **I am sometimes** able **to** develop a rapport in a healthcare setting | **I am rarely able to** develop a rapport in a healthcare setting |
| Comments | Likert Scale 6 5 4 3 2 1 | | | |
| **Which statement best describes your ability to use presumptive statement(s) around vaccination during a clinical encounter?** | - I assume a person wants to optimize their personal health outcomes - I assume a person will choose to get immunized - I am able to give strong recommendations - I am able to make the presumptive statement in a relevant and appropriate manner - I am respectful, calm, and non-judgmental | **I am consistently able to** use appropriate presumptive statement techniques | **I am sometimes able to** use presumptive statement techniques | **I am rarely able to** use presumptive statement techniques |
| Comments | Likert Scale 6 5 4 3 2 1 | | | |
| **Which statement best describes how confident you are using interviewing techniques during a clinical encounter to help identify a patient’s point of view and reasons for vaccine hesitancy?** | - I can affirm a patient’s point of view - I can express and show empathy towards patients - I can support and identify discrepancies (between patient’s wants and actions) - I can effectively manage resistance - I can support patient’s self-efficacy | **I am fully confident that I can** use motivational interview strategies to identify reasons for vaccine hesitancy. | **I am somewhat confident that I can** use motivational interview strategies to identify reasons for vaccine hesitancy. | **I am not confident that I can** use motivational interview strategies to identify reasons for vaccine hesitancy. |
| Comments | Likert Scale 6 5 4 3 2 1 | | | |
| **Which statement best describes how confident you are identifying the root cause or pattern behind vaccine hesitancy during a clinical encounter to ensure a personalized approach to the conversation?** | - I can identify patient priorities vs healthcare provider priorities - I can communicate in a sensitive manner - I can involve patients and/or families in conversations | **I am fully confident that I can** identify the root cause of vaccine hesitancy during patient conversations. | **I am somewhat confident that I can** identify the root cause of vaccine hesitancy during patient conversations. | **I am not confident that I can** identify the root cause of vaccine hesitancy during patient conversations. |
| Comments | Likert Scale 6 5 4 3 2 1 | | | |
| **Which statement best describes how confident you are supporting patient decision making at the conclusion of the conversation to develop a personalized and achievable plan for vaccination?** | - I can support patients to recognize and acknowledge risks and benefits of each option - I can support clients’ values and preferences - I can address patient needs - I can provide additional resources if requested | **I am fully confident that I can** incorporate strategies that meet the patient’s priorities and needs to establish a personalized and achievable plan for vaccination. | **I am somewhat confident that I can** incorporate strategies that meet the patient’s priorities and needs to establish a personalized and achievable plan for vaccination. | **I am not confident that I can** incorporate strategies that meet the patient’s priorities and needs to establish a personalized and achievable plan for vaccination. |
| Comments | Likert Scale 6 5 4 3 2 1 | | | |

1. VSG 3 - Self-regulation Assessment Rubric

| **Learning Outcome** | **Confidence/Self-efficacy Indicators** |  |  |  |
| --- | --- | --- | --- | --- |
| **Which statement best describes how confident you are engaging in therapeutic communication while building trust and collaborating effectively with a patient or caregiver?** | - I can model respectful conversations - I can identify and apply appropriate communication techniques - I can explore reasons behind a caregiver's perspective (e.g. fixed beliefs, misinformation) - I can utilize and model a strength-based approach | **I am fully confident that I can** engage in therapeutic communication while building trust and collaborating effectively | **I am somewhat confident that I can** engage in therapeutic communication while trying to build trust and collaborate effectively | **I am not confident that I can** engage in therapeutic communication or attempt to build trust and collaborate |
| Comments: | Likert Scale 6 5 4 3 2 1 | | | |
| **Which statement best describes how confident you are guiding a difficult conversation with a patient or caregiver in order to preserve the therapeutic relationship, and maintain focus on the patient’s health needs?** | - I can ask open ended questions and foster a sense of trust and curiosity - I can avoid having a judgmental attitude towards patients - I can be flexible - I can constructively establish mutually acceptable goals - I can shift the conversation in constructive ways while validating patient perspectives | **I am fully confident that I can** guide a difficult conversation with a caregiver | **I am somewhat confident that I can** guide a difficult conversation with a caregiver | **I am not confident that I can** guide a difficult conversation with a caregiver |
| Comments: | Likert Scale 6 5 4 3 2 1 | | | |
| **Which statement best describes your ability to foster personal resilience in the face of moral distress during and after a difficult conversation?** | - I am able to identify factors that contribute to a difficult conversation (opposing views, high emotion) - I am able to maintain an open attitude - I am able to acknowledge the presence of strong emotions in myself - I am able to self-regulate my personal responses - I am able to maintain respectful conversations - I am able to recognize the presence of moral distress - I am able to identify when core values are being opposed during conversation | **I am consistently able to** foster personal resilience in the face of moral distress during and after a difficult conversation | **I am sometimes able to** foster personal resilience in the face of moral distress during and after a difficult conversation | **I am rarely able to** foster personal resilience in the face of moral distress during and after a difficult conversation to |
| Comments: | Likert Scale 6 5 4 3 2 1 | | | |
| **Which statement best describes your ability to maintain a professional sense of self-efficacy after a challenging interaction?** | - I am able to acknowledge and normalize interactions that cause personal stress - I am able to engage in self-kindness instead of self-judgment - I am able to engage in mindfulness and self-compassion | **I am consistently able to** maintain a professional sense of self-efficacy after a challenging interaction | **I am sometimes able to** maintain a professional sense of self-efficacy after a challenging interaction | **I am rarely able to** maintain a professional sense of self-efficacy after a challenging interaction |
| Comments | Likert Scale 6 5 4 3 2 1 | | | |
