# Supplementary material for: Development and evaluation of virtual simulation games to increase the confidence and self-efficacy of healthcare learners in vaccine communication, advocacy, and promotion
Source: BMC Med Educ. 2024 Feb 25;24:190. doi: 10.1186/s12909-024-05169-9 (PMC10895736; doi:10.1186/s12909-024-05169-9)
Supplement: Supplementary file 2 — Supplementary Material 2: Additional file 2. VSG self-assessment rubrics (VSG 1, VSG 2, VSG 3). [file 12909_2024_5169_MOESM2_ESM.docx]

**Additional file 4. Pre and Post Intervention Surveys**

1. Pre-Intervention Survey
2. Please enter your age: ____
3. Which of the following best describes you?

- Woman
- Man
- Non-binary
- Two-spirit
- Other (describe):

1. What program are you enrolled in?

- Pharmacy
- Nursing
- Medicine
- Other

1. What year of your program are you currently enrolled in?
   - 1st
   - 2nd
   - 3rd
   - 4th
   - Other
2. If you are a medical resident, what is your specialty?
   1. Internal medicine (IM)
   2. Family medicine (FM)
   3. Obstetrics and Gynecology (OBGYN)
   4. Pediatrics (Peds)
   5. Emergency Medicine (EM)
   6. Other (please specify)
3. Have you ever had a vaccine conversation with a patient? (Not necessarily about vaccine hesitancy)

- Yes
- No

1. Did you learn about how to have **vaccine conversations with patients** in your program?
   - Yes
   - No
2. If yes, please select how/when you learned about vaccine conversations from the list below:
   1. Theory / coursework
   2. Lab/simulation setting
   3. Clinical practice
   4. Workshop
   5. Other
3. Post Intervention Survey
4. While some of the VSGs are targeted to COVID-19 vaccines, do you think they would still be relevant to discussing other routine immunizations with patients? How could this be applicable to other vaccine conversations?
5. Please provide any general feedback about the online learning module below. Were there things you liked or disliked? Please describe any changes or things you would’ve liked to have been done differently.
6. Are you interested in participating in a Focus Group after the intervention? If yes, please ensure you completed both the pre and post intervention survey and self-assessments, and all 3 VSGs.
