# Supplementary material for: Development and evaluation of virtual simulation games to increase the confidence and self-efficacy of healthcare learners in vaccine communication, advocacy, and promotion
Source: BMC Med Educ. 2024 Feb 25;24:190. doi: 10.1186/s12909-024-05169-9 (PMC10895736; doi:10.1186/s12909-024-05169-9)
Supplement: Supplementary file 3 — Supplementary Material 3: Additional file 3. Decision point map and rationale example (VSG 1). [file 12909_2024_5169_MOESM3_ESM.docx]

**Additional file 3.** Decision Point Map and Rationale Example (VSG 1)

Game Title: Team 1- Booster

Learning Objectives

1. Regulates own emotions before, during and after a clinical encounter to promote a therapeutic relationship with patients
2. Develop a rapport in a healthcare setting to build ongoing relationship with the patient
3. Make presumptive statements around vaccination during a clinical encounter with a patient who has not completed a vaccine series to reinforce positive behaviours
4. Recognize vaccine hesitancy profile during a routine clinical encounter to determine approaches to understanding reasons for not continuing with vaccine series
5. Support decision-making for patient who has not completed a vaccine series to develop a personalized and achievable plan for vaccination

| Decision Point | Scene | Question | Response 1  (Correct) | Response 2  (Incorrect) | Response 3  (Incorrect) |
| --- | --- | --- | --- | --- | --- |
| 1 | HCP accompanied by a student is reviewing electronic chart on computer/ laptop outside door of clinic room. HCP points to laptop screen (vaccine series box is highlighted) and HCP points out and says to the student that booster is overdue. | As the HCP how would you prepare to address a conversation about the vaccine booster? | Review personal biases regarding vaccines  Rationale:  Important not to let personal biases influence informing the patient. The goal is to provide a safe environment for this conversation to improve patient outcomes. | Assume patient is vaccine hesitant  Rationale:  Important not to make assumptions as this interferes with the therapeutic relationship. The goal is to provide a safe environment for this conversation to improve patient outcomes. | Avoid engaging in conversation about vaccines  Rationale:  Avoiding and important health outcome (appropriate vaccination) is not providing the best patient care. The goal is to provide a safe environment for this conversation to improve patient outcomes. |
| 2 | HCP speaking to student: I’d like the patient to get their booster (third dose of vaccine). It is recommended for persons with an underlying condition such as diabetes to have all their vaccines up to date. Let’s address this today.  Knock and enter clinic room. Introduce themselves. | What would the HCP say to initiate the conversation about the vaccine? | “How are you doing today? Did you do anything fun over the weekend?”  Rationale: It is important to remain nonjudgmental and establish a rapport with the patient prior to initiating a sensitive conversation. The goal is to provide a therapeutic environment to promote a longitudinal, trusting relationship | “Can I ask why you haven’t received your third dose/booster of the COVID vaccine yet?”  Rationale:  This is a judgmental statement. It is important to remain nonjudgmental and establish a rapport with the patient prior to initiating a sensitive conversation. The goal is to provide a therapeutic environment to promote trust. | “I notice you’re not up to date on some of your vaccines. I worry that people with diabetes are at a higher risk for ending up in ICU if they’re not vaccinated”  This statement assumes a lack of knowledge and provides knowledge in an overwhelming manner. It is important to remain nonjudgmental and establish a rapport with the patient prior to initiating a sensitive conversation. The goal is to provide a therapeutic environment to promote trust |
| 3 | Engage with the patient to develop rapport before addressing vaccine discussion. | How would the HCP initiate the conversation about the vaccine? | “When are you booked to have your COVID booster?  Rationale: The first step of the PrOTCT model is to make a presumptive statement. | “Are you interested in receiving a 3rd dose of the COVID vaccine?  Rationale:  This is a participatory statement. | “You need to get your COVID booster. The student can give it to you today”  Rationale: Presumptive statements must be carefully worded not to be judgemental. This strongly worded and judgemental statement impairs further conversation and damages the patient-provider relationship. |
| 4 | When are you booked…  Patient: I’ve been putting it off because I was fatigued and felt sick for two days after the second dose. | How would the HCP respond? | Offer to provide information about vaccine side effects and how to manage them  Rationale: This response aligns with the second step of the PrOTCT model and addresses the patient’s concerns and provides them with potential solutions to promote best health outcomes | Provide information about diabetes and its relationship to COVID.  Rationale:  This response does not address the underlying cause of the vaccine hesitancy. | Share personal experience with the COVID vaccine  Rationale: This response minimizes the patient’s experience with the vaccine |
| 5 | Can I provide some information about the vaccine for you…”  Patient: agrees  HCP provides information | What would the HCP do next? | Ask about concerns  Rationale: This response aligns with the PrOTCT model to allow the discussion to be tailored to the patient and specific to their concerns. | Book a Friday appointment  Rationale: This response is presumptive and does not address the patient’s specific concerns | Suggest patient book an appointment  Rationale: This response is passive and least likely to result in action by the patient |
| 6 | HCP asks do you have any specific concerns. Pt acknowledges the information provided. But is it worth these side effects? I don’t think I need another dose. | How would the HCP respond? | “The reason I think you should get a third dose is because we now know that two doses doesn’t provide the best protection, especially since you have diabetes.”  Only address the importance of receiving a third dose of the vaccine.  Rationale: the counselling is tailored to the patient and the patient’s specific concerns which aligns with the PrOTCT model. | “Let me tell you more about COVID.”  Provide information about COVID, vaccine importance, vaccine side effects  Rationale: this response is overwhelming and not specific to the patient’s concerns. | “Public health recommends that everyone gets a third dose.”  Reinforce the need for receiving the third dose  Rationale: this response is not tailored to the patient or the patient’s specific concerns. It does not provide rationale to come to a shared understanding. |
| 7 | Patient: “I didn’t realize there was such a big difference between two doses and three doses…and maybe I should be more concerned now that I have diabetes. | How would the HCP respond? | “I’m happy I was able to provide some helpful information. It sounds like you’re motivated. I can help you book your vaccine appointment now.  Rationale: this response provides positive reinforcement and makes an actionable plan which aligns with the final step of the PrOTCT model. | “I’m glad to hear we’re on the same page. Hopefully when we see you again in three months you will have had your third dose.”  Rationale: this response provides positive reinforcement but does not include an actionable plan. | “Now that I’ve provided you with information. You can check with your family doctor at your follow-up appointment to help you decide.”  Rationale: All HCPs have an equal responsibility to advocate for vaccines. |
| 8 | I’m happy to …book your appointment. Let’s book you for Friday and you’ll have the weekend to recover if you have any side effects.  Patient: Thank you.  Fade to black  HCP and student in the room to debrief. Role model debriefing…how I felt during, how I took a pause and reoriented, debriefing is important… |  |  |  |  |
