# Supplementary material for: Development and evaluation of virtual simulation games to increase the confidence and self-efficacy of healthcare learners in vaccine communication, advocacy, and promotion
Source: BMC Med Educ. 2024 Feb 25;24:190. doi: 10.1186/s12909-024-05169-9 (PMC10895736; doi:10.1186/s12909-024-05169-9)
Supplement: Supplementary file 4 — Supplementary Material 4: Additional file 4. Pre- and post-intervention surveys. [file 12909_2024_5169_MOESM4_ESM.docx]

**Additional file 1****.** Learning Outcomes and Indicators Example (VSG 1)

| **VSG 1** | | | |
| --- | --- | --- | --- |
|  | **DO WHAT** | **WHERE/WHEN** | **WHY** |
| 1  confidence | Self-regulation  Regulates own emotions | before, during and after a clinical encounter | to promote a therapeutic relationship with patients |
| 2  confidence | Presumptive statement  Make presumptive statements around vaccination | during a clinical encounter with a patient who has not completed a vaccine series | to reinforce positive behaviours |
| 3  Self-efficacy/ ability | Pattern recognition  Recognize vaccine hesitancy profile | during a routine clinical encounter | to determine approaches to understanding reasons for not continuing with vaccine series |
| 4  Self-efficacy/ ability | Communication  Support decision-making | for patient who has not completed a vaccine series | to develop a personalized and achievable plan for vaccination |
